# The Clinical Effect of Xylazine Premedication in Water Buffalo Calves (*Bubalus bubalis*) Undergoing Castration under General Anaesthesia

**DOI:** 10.3390/ani11123433

**Published:** 2021-12-01

**Authors:** Giulia Guerri, Ilaria Cerasoli, Paola Straticò, Ippolito De Amicis, Brunella Giangaspero, Vincenzo Varasano, Andrea Paolini, Augusto Carluccio, Lucio Petrizzi

**Affiliations:** 1 Faculty of Veterinary Medicine, University of Teramo, Località Piano D’Accio, 64100 Teramo, Italy; guerri.giu@gmail.com (G.G.); ideamicis@unite.it (I.D.A.); brunellagiangaspero@gmail.com (B.G.); vvarasano@unite.it (V.V.); apaolini@unite.it (A.P.); acarluccio@unite.it (A.C.); lpetrizzi@unite.it (L.P.); 2 Clinica Veterinaria Borghesiana, Via di Vermicino 96, 00133 Roma, Italy; ilaria.cerasoli@gmail.com

**Keywords:** buffaloes, castration, anaesthesia, xylazine, ketamine, isoflurane

## Abstract

**Simple Summary:**

Castration is used to improve the management of buffalo calves and their meat quality. Despite the growing interest in livestock welfare, water buffaloes have not been studied as frequently as other species. General anaesthesia produces complete unconsciousness, insensitivity to pain and muscle relaxation. The quality and safety of general anaesthesia may be improved using sedatives prior to the induction of general anaesthesia. The aim of this study was to evaluate the effect of two different intramuscular doses of xylazine for premedication, followed by ketamine induction, in water buffalo calves undergoing isoflurane maintenance anaesthesia for routine castration surgery. Based on the results obtained, we can conclude that both protocols are safe for buffalo castration, but the higher dose protocol is recommended for buffalo orchiectomy.

**Abstract:**

Castration is one of the most frequently performed surgical procedures in livestock. All methods of castration are associated with a significant degree of pain, affecting the patients’ welfare. Limited information on species-specific protocols in buffaloes is available. Considering the importance of this species and the scarcity of reports available, the objective of this study was to evaluate the effects of two different intramuscular doses of xylazine for premedication in water buffalo calves undergoing isoflurane maintenance anaesthesia for routine castration surgery. Twenty clinically healthy male water buffaloes undergoing bilateral orchiectomy were randomly assigned to two groups of 10 animals each: Group 1 was premedicated with xylazine 0.1 mg/kg bw i.m. and Group 2 was premedicated with xylazine 0.15 mg/kg bw i.m. Anaesthesia was induced by ketamine (2 mg/kg bw i.v.) and maintained with isoflurane-oxygen-air in both groups. The HR, RR, presence/absence of ataxia, sedation, depth of anaesthesia, muscle relaxation status, response to acoustic and tactile stimuli, eyelid reflex, extent of salivation and stimulus were evaluated every 5 min. Both protocols provided consistent sedation and were safe. Faster and more satisfactory sedation was recorded when xylazine was administered at 0.15 mg/kg bw i.m., leading us to recommend this dose for buffalo orchiectomy.

## 1. Introduction

Water buffalo (*Bubalus bubalis*) is a valuable species with excellent zootechnical characteristics for both milk and meat production [1]. As a result of the growing economic interest, buffalo farming moved from traditional extensive techniques to more intensive systems [2,3]. Such changes increased the expression of undesired behaviours, such as excessive aggressive interactions, negatively affecting buffalo behaviour, welfare and production efficiency [1,2].

The castration of water buffalo males is performed to improve their management, reduce aggressive and sexual behaviour, and improve meat quality [4,5]. Despite the beneficial effects, all methods of castration produce physiological, neuroendocrine and behavioural changes indicative of pain and distress [6,7]. Unmitigated pain may suppress immune function and increase susceptibility to disease, not only compromising welfare but also increasing the risk of morbidity and mortality [8,9]. The growing global concern about pain related to routine husbandry procedures such as castration, dehorning and tail docking challenges researchers to develop effective methods to relieve pain and stress in livestock [6,7,8,9,10].

Concerning these procedures, water buffaloes have not been addressed as frequently as other species [4]. Because the anatomical and physiological characteristics of cows are similar to those of buffaloes, buffaloes are often considered and managed as cattle. However, species-specific features need to be considered in anaesthesia and analgesia, and specific dosing protocols are mandatory, especially in animals intended for human consumption [10,11].

Alpha_2_-adrenoceptor agonist agents are widely used as anxiolytics, analgesics, central muscle relaxants and preanesthetic sedatives in large ruminants [12,13]. Xylazine is widely used in cattle which are more sensitive to the effects of this drug and require one-tenth of the dose used in horses [14]. In buffaloes, high doses of xylazine cause marked sedation, increased salivation, regurgitation and bradycardia [15]. However, a lower intramuscular dose (0.10 to 0.15 mg/kg) was suggested in buffalo calves, suggesting that it may have potential for use as a premedication in these species [16].

Considering the paucity of information about this species, the objective of the present study was to evaluate and compare the effect of two different intramuscular doses of xylazine for premedication, followed by ketamine induction, in water buffalo calves undergoing isoflurane maintenance anaesthesia for routine castration surgery.

## 2. Materials and Methods

### 2.1. Animals

The study protocol was approved by the local ethical committee (O.p.B.A. of the Istituto Zooprofilattico of Teramo “G. Caporale”, Prot.2021/0000338/GEN/GEN). Healthy male water buffalo calves aged 2 months-1 year who were presented for castration at the Veterinary Teaching Hospital (VTH) of the University of Teramo were prospectively recruited (ASA I, American Society of Anesthesiologists). Buffalo owners were aware of the procedure that was going to be undertaken and signed an informed consent form. Cattle were weighed and monitored on arrival at the clinic. To allow animals to adapt to the new environment prior to baseline behavioural assessment, they were maintained for 48 h in a stall with free access to feed and water.

### 2.2. Experimental Protocols

After the baseline behavioural assessment, the calves were fasted for 12 h, and water was withheld for 8 h prior to anaesthesia. The calves were randomly divided by flipping a fair coin into two different groups: the animals assigned to Group 1 received xylazine (Nerfasin^®^; A.T.I. Srl, Ozzano Emilia, Italy) at a dose of 0.1 mg/kg bw i.m. (intramuscularly); those assigned to Group 2 received xylazine at a dose of 0.15 mg/kg bw i.m. Once buffaloes reached sternal recumbency, the jugular groove was clipped and evaluated ultrasonographically (Logiq S8XD Clear, GE Healthcare, Little Chalfont, UK; linear probe 8.5–10 MHz) in three portions of the neck (proximal, middle and distal) to allow ultrasound-guided catheterisation of the vein. A subcutaneous inoculation of 1 mL of Lidocaine 2% (Lidocaine 2%^®^, Ecuphar Italia Srl, Milano, Italy) was performed, and after 5 min, a venous catheter (14 G, 160 mm, Secalon-T^TM,^ Merit Medical, Singapore) was inserted into the jugular vein under ultrasonographic guidance. If jugular catheterisation failed after 4 attempts, a venous catheter (22 G 25 mm, Surflo^TM^ I.V. Catheter, Terumo Italia Srl, Rome, Italy) was inserted into the marginal vein of the ear. The number of attempts, the time taken to reach sternal recumbency and the reaction to catheterisation at different times (the trichotomy of the area, application of isopropyl alcohol, disinfection of the area and cutting, insertion and fixation of the venous catheter) were recorded.

The induction of anaesthesia was obtained by an intravenous administration of ketamine (Ketavet 100^®^; ACME Srl, Reggio Emilia, Italy) (2 mg/kg bw). If the degree of muscle relaxation and hypnosis were not sufficient to allow orotracheal intubation, an additional dose of xylazine (0.025 mg/kg bw) was administered intravenously. In particularly alert calves, an additional dose of ketamine (0.25 mg/kg bw i.v.) was administered. All additional boluses were recorded. After induction, guided intubation with a mouth opener and a stylet was performed, and the calves were placed in dorsal recumbency on the surgical table. Anaesthesia was maintained with isoflurane (Isoflo^®^, Esteve SpA, Milano, Italy) (MAC 1–1.2%) in oxygen and air (1:2 ratio) through a circular anaesthetic system (VML Anesthesia Machine; MDS Matrx, New York, NY, USA).

### 2.3. Evaluation of Sedation

The basal rectal temperature, heart rate (HR) and respiratory rate (RR) were measured and recorded before the administration of any drug.

**Figure 1:**
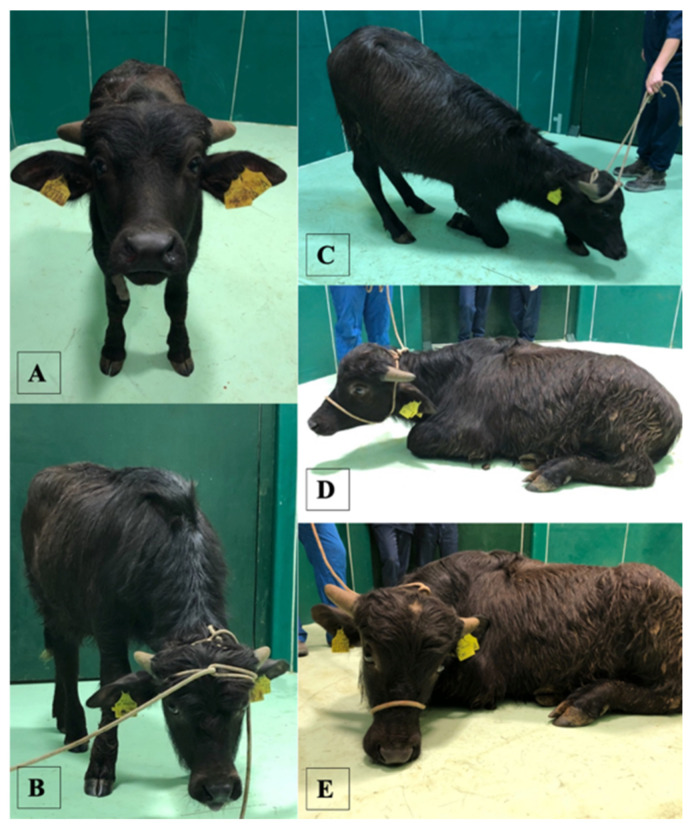
Different degrees of sedation and ataxia in water buffaloes in our study. (**A**) no sedation, buffalo alert, eyes open, no ataxia; (**B**) mild sedation, drooping of eyelids, mild sensory and motor deficit; (**C**) moderate sedation, drooping of eyelids, moderate sensory and motor deficit, knuckling of the fetlocks; (**D**,**E**) strong sedation, drooping of eyelids, sensory and motor deficit, assuming recumbency.

The calves of both groups were evaluated at T0 (premedication with xylazine), 2 and 5 min after and every 5 min from the first administration of xylazine. The HR, RR, presence/absence of ataxia, sedation, depth of anaesthesia, muscle relaxation status, response to acoustic and tactile stimulus, eyelid reflex and extent of salivation were evaluated by an external observer who was blinded to the treatment received by each calf (Table 1; Figure 1) [17,18]. Moreover, the presence/absence of regurgitation, tympanism and reflux was recorded.

### 2.4. Surgery

Flunixin meglumine (Flunifen^®^; Ceva Santé Animale, Libourne, France) (1.1 mg kg bw I.V.) and benzylpenicillin + dihydrostreptomycin (Repen^®^; FATRO S.p.A., Bologna, Italy) (9000–12,000 UI/kg bw I.M.) were administered just before the beginning of surgery.

Once under general anaesthesia, trichotomy and the disinfection of the surgical area were performed. A 22 G 22 mm catheter (Surflo^TM^ I.V. Catheter, Terumo Italia Srl, Rome, Italy) was placed in the auricular artery to monitor invasive arterial blood pressure throughout the surgical procedure. Furthermore, after surgical preparation, an intrafunicular block was performed with 10 mL of lidocaine 2% for each testicle [9,19].

Ten minutes after local anaesthesia, bilateral orchiectomy with a closed technique was performed by an experienced surgeon [20].

Throughout the surgical procedure, the buffaloes were monitored continuously, and data recorded every five minutes (GE 850 Anaesthesia Monitor, GE Healthcare, Chalfont St Giles, UK; Dura-Cuf^®^ 12–19 cm; Critikon Blood Pressure Cuffs, GE Healthcare, Chalfont St Giles, UK). For electrocardiography (ECG), a standard base apex lead electrocardiogram was performed. Moreover, once the arterial catheter was placed, blood gas analysis was performed (Abaxis VetSCan i-Stat^®^1, GE Healthcare, Chalfont St Giles, UK) and repeated after 30 min in case the surgical procedure was still ongoing to assess the acid–base balance [21]. The duration of the surgical procedure, anaesthesia, and eventual intra- and postoperative complications were recorded. Once the surgery was completed (at the removal of the Backhaus forceps from the surgical field), the calves were disconnected from the anaesthetic circuit and moved to the recovery box.

### 2.5. Recovery

The time to extubation and sternal recumbency, the number of attempts and the time to the standing position were recorded. The final recovery score was calculated at the end of the procedure based on the scoring system modified from Alonso et al. 2020, from 1 to 6 (1 = one attempt, little to no ataxia; 2 = 1 or 2 attempts, some ataxia; 3 = > 2 attempts, quiet recovery; 4 = > 2 attempts, moderate recovery; 5 = > 2 attempts, excitation; 6 = very bad recovery, high risk of injury) [22].

After the procedure, the animals were kept in the same enclosure in which they had been housed prior to surgery. After surgery, according to the UNESP-BOTUCATU unidimensional composite pain scale for assessing postoperative pain in cattle [23], patients were monitored at 30, 60 and 90 min and then at 6-, 12- and 24-h intervals. Locomotion, interactive behaviour, activity, appetite and other miscellaneous behaviours were analysed and scored, and rescue analgesics were administered if the cut-off value of final total score of ≥5 was reached.

### 2.6. Statistical Analysis

All statistical analyses were performed using SPSS V.27.0, IBM. A Mann–Whitney U test was used to compare the values obtained between the two experimental groups. Values of *p* < 0.05 were considered significant. Data are presented as the mean and standard deviation (SD).

## 3. Results

Twenty male water buffalo calves were included in the study; 10 were assigned to Group 1 and 10 were assigned to Group 2. No significant difference in age was found between the groups (*p* = 0.16) (Table 2). Conversely, the mean body weight in Group 1 was significantly higher than that in Group 2 (*p* = 0.002) (Table 2). In Group 1, the mean catheterisation time was significantly longer, and a significantly higher number of attempts was necessary to perform the catheterisation (*p* = 0,01; *p* = 0.01) (Table 2). Moreover, in Group 2, the catheterisation of the jugular vein was performed in all the buffalos, whereas in Group 1, the catheterisation of the jugular vein failed in four buffalos; thus, the marginal vein of the ears was catheterised. Buffalos in Group 1 showed a significantly higher mean intubation time (*p* = 0.03) (Table 2). A significant difference in endotracheal tube (ET) size was also found between groups, with a smaller ET used in Group 2 (*p* = 0.003). When analysing the number of top-ups of xylazine and ketamine, no significant differences were observed between the groups (*p* = 0.50; *p* = 0.38) (Table 2).

The mean duration of anaesthesia and mean surgical time were not significantly different between groups, although they were longer for Group 1 (*p* = 0.16; *p* = 0.17) (Table 3). No intraoperative complications were recorded in Group 2; however, in Group 1, one buffalo presented reflux 25 min after starting general anaesthesia. No postoperative complications were recorded in Group 1; however, one buffalo in Group 2 died 24 h postoperatively because of tympanism. No significant difference was found in the time to extubation, time to reach sternal recumbency or time to standing, although the time was longer for Group 2 (*p* = 0.08; *p* = 0.07; *p* = 0.30) (Table 3; Figure 1). When considering the final recovery score, no significant difference between groups was observed, although the final recovery score was higher in Group 2 (*p* = 0.16) (Table 3).

### 3.1. Heart Rate (HR) and Respiratory Rate (RR)

**Figure 2:**
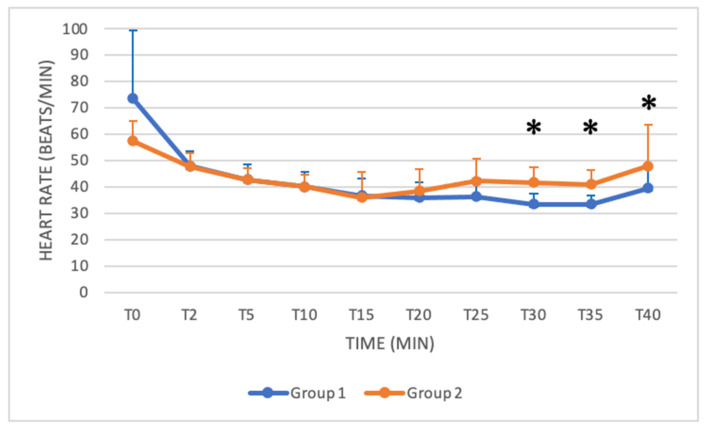
Mean HR from T0 to T40 compared in Group 1 (blue line) and Group 2 (orange line) (*: *p* < 0.05). The figure has different checkpoints (T0, T2, T5, etc.) and is integrated with lines at each checkpoint, indicating the SD (standard deviation).

The mean HR at baseline (BL) was 63.30 ± 15.80, and the mean RR at BL was 20.90 ± 5.52. In both groups, HR and RR decreased over time. After sedation, the mean HR was slightly higher in Group 1 from T0 to T15, becoming significantly lower in Group 1 from T30 to T40 (Table 4 and Figure 2). No significant difference in RR was found between groups (Table 4).

### 3.2. Sedation (SDX) and Ataxia (ATX)

**Figure 3:**
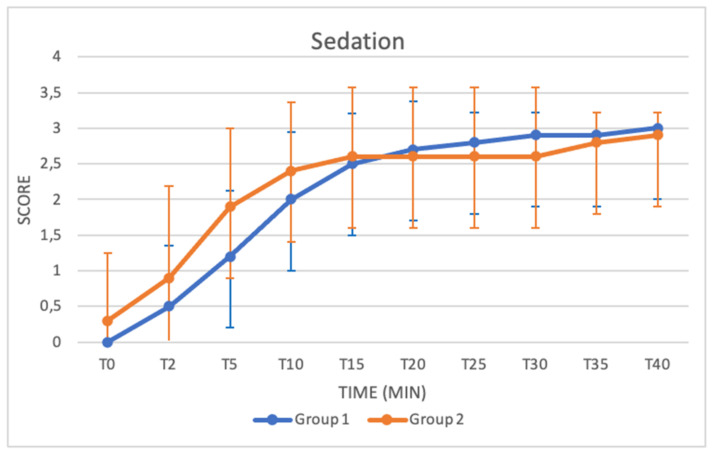
Trend in sedation from T0 to T40 compared in Group 1 (blue line) and Group 2 (orange line). The figure has different checkpoints (T0, T2, T5, etc.) and is integrated with lines at each checkpoint, indicating the range.

**Figure 4:**
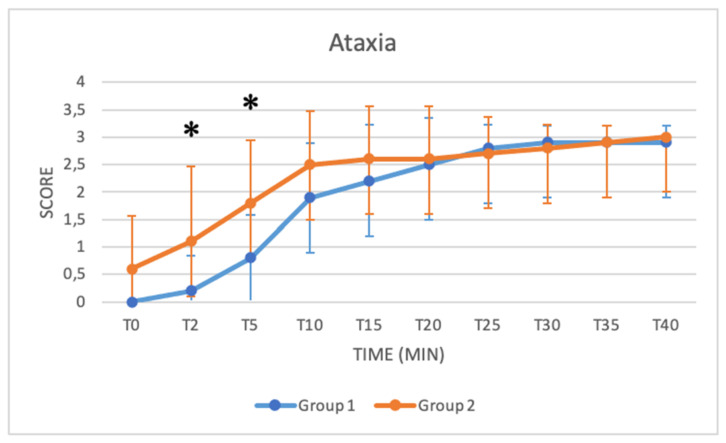
Trend in ataxia score from T0 to T40 compared in Group 1 (blue line) and Group 2 (orange line) (*: *p* < 0.05). The figure has different checkpoints (T0, T2, T5, etc.) and is integrated with lines at each checkpoint, indicating the range.

Sedation and ataxia in both groups were recorded from T0 to T40. Both sedation and ataxia scores were 0 at T0 in Group 1, whereas in Group 2, the sedation score was 0.3 ± 0.94, and the ataxia score was 0.7 ± 1.15. Both values increased over time, and the ataxia score reached its maximum at T40 in both groups. Statistically higher ataxia scores were found at T2 and T5 in Group 2 (T2: Group 1: 0.20 ± 0.63; Group 2: 1.10 ±1.37) (T5: Group 1: 0.80 ± 0.78; Group 2: 1.80 ± 1.13) (*p* = 0.03; *p* = 0.03) (Figure 3 and Figure 4).

### 3.3. Schemes Modified from Singh

**Figure 5:**
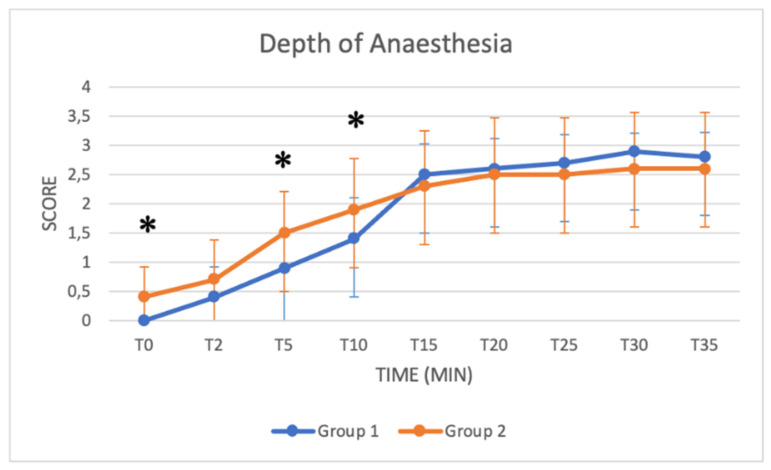
Line graph showing the depth of anaesthesia (*y* axis) as a function of time (*x* axis) from T0 to T35 compared in Group 1 (blue line) and Group 2 (orange line) (*: *p* < 0.05). The figure has different checkpoints (T0, T2, T5, etc.) and is integrated with lines at each checkpoint, indicating the range.

**Figure 6:**
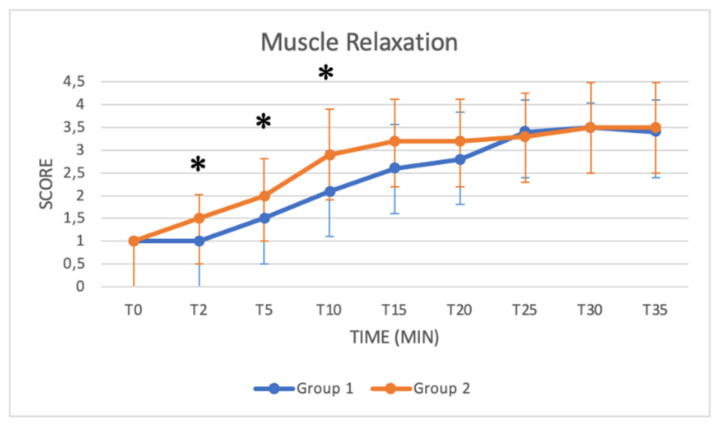
Line graph showing the muscle relaxation score (*y* axis) as a function of time (*x* axis) from T0 to T35 compared in Group 1 (blue line) and Group 2 (orange line) (*: *p* < 0.05). The figure has different checkpoints (T0, T2, T5, etc.) and is integrated with lines at each checkpoint, indicating the range.

**Figure 7:**
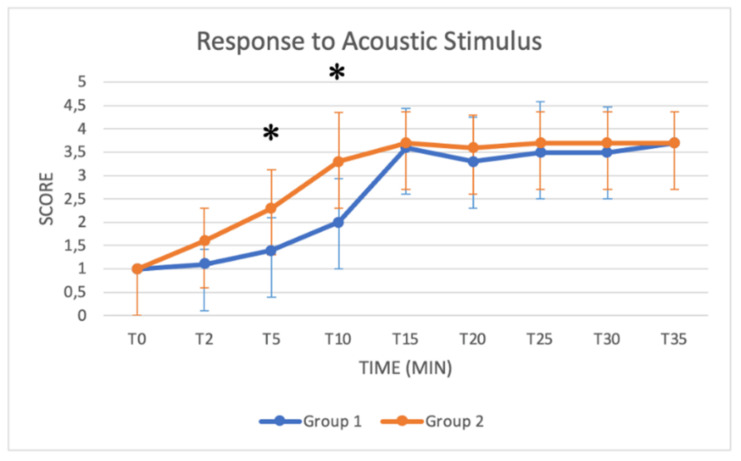
Line graph showing the response to acoustic stimulus score (*y* axis) as a function of time (*x* axis) from T0 to T35 compared in Group 1 (blue line) and Group 2 (orange line) (*: *p* < 0.05). The figure has different checkpoints (T0, T2, T5, etc.) and is integrated with lines at each checkpoint, indicating the range.

**Figure 8:**
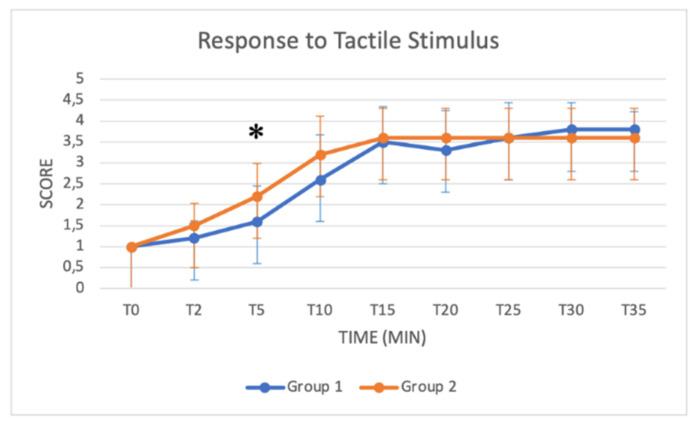
Line graph showing the response to tactile stimulus score (*y* axis) as a function of time (*x* axis) from T0 to T35 compared in Group 1 (blue line) and Group 2 (orange line) (*: *p* < 0.05). The figure has different checkpoints (T0, T2, T5, etc.) and is integrated with lines at each checkpoint, indicating the range.

**Figure 9:**
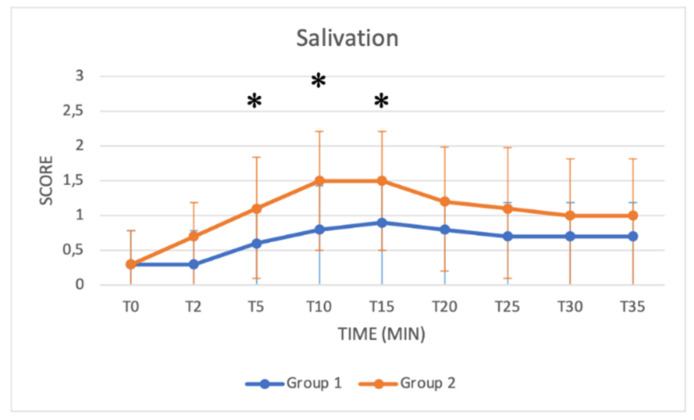
Line graph showing the salivation score (*y* axis) as a function of time (*x* axis) from T0 to T35 compared in Group 1 (blue line) and Group 2 (orange line) (*: *p* < 0.05). The figure has different checkpoints (T0, T2, T5, etc.) and is integrated with lines at each checkpoint, indicating the range.

These schemes include several key parameters for monitoring sedation from T0 to T35: the depth of anaesthesia, muscle relaxation status, response to acoustic and tactile stimulus, eyelid reflex and extent of salivation. Median values increased in both groups as a function of time (Figure 5, Figure 6, Figure 7, Figure 8 and Figure 9). Statistical differences between groups were found in the depth of anaesthesia, muscle relaxation, and response to acoustic and tactile stimuli at T5 and T10, with higher values in Group 2 (Table 5, Figure 5, Figure 6, Figure 7 and Figure 8).

The *salivation* score was significantly higher in Group 2 at T5, T10 and T15 (*p* = 0.03; *p* = 0.01; *p* = 0.02) (Table 5; Figure 9). No significant difference was found in the evaluation of *eyelid reflex* scores between groups (Table 5). In Group 1, no buffalos presented reflux/regurgitation or tympanism, whereas in Group 2, a buffalo presented reflux at T30 (Table 5). In both groups, 3/10 buffaloes showed mild regurgitation, and 3/10 buffaloes in Group 2 showed mild tympanism at the end of or after surgery in the recovery box.

### 3.4. Arterial Blood Pressure

**Figure 10:**
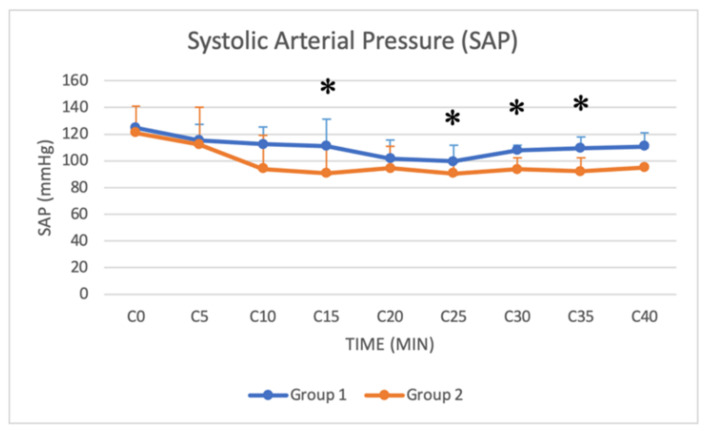
Line graph showing SAP (*y* axis) as a function of time (*x* axis) from C0 to C40 compared in Group 1 (blue line) and Group 2 (orange line) (*: *p* < 0.05). The figure has different checkpoints (T0, T2, T5, etc.) and is integrated with lines at each checkpoint, indicating the SD (SAP: systolic arterial pressure; SD: standard deviation).

**Figure 11:**
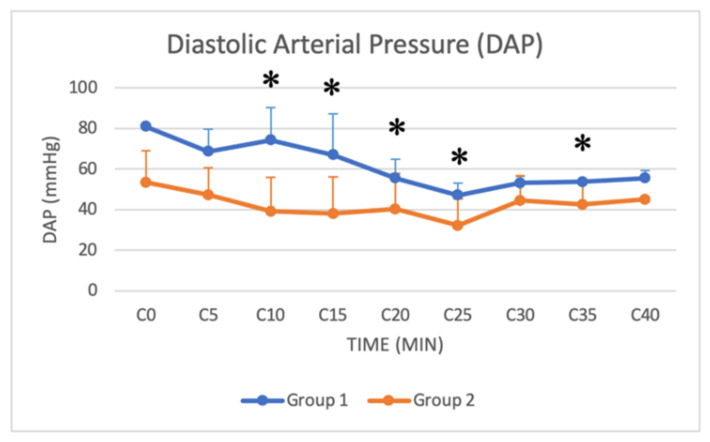
Line graph showing DAP (*y* axis) as a function of time (*x* axis) from C0 to C40 compared in Group 1 (blue line) and Group 2 (orange line) (*: *p* < 0.05). The figure has different checkpoints (T0, T2, T5, etc.) and is integrated with lines at each checkpoint, indicating the SD (DAP: diastolic arterial pressure; SD: standard deviation).

**Figure 12:**
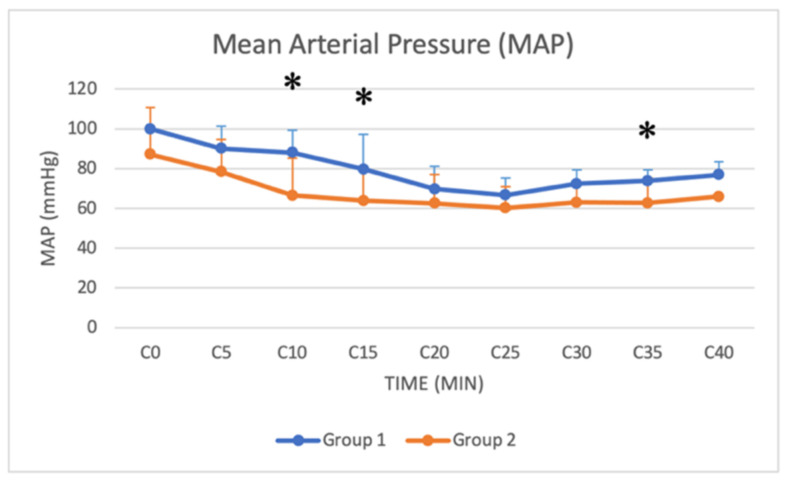
Line graph showing MAP (*y* axis) as a function of time (*x* axis) from C0 to C40 compared in Group 1 (blue line) and Group 2 (orange line) (*: *p* < 0.05). The figure has different checkpoints (T0, T2, T5, etc.) and is integrated with lines at each checkpoint, indicating the SD (MAP: mean arterial pressure; SD: standard deviation).

Systolic, mean and diastolic arterial pressure (SAP, MAP and DAP, respectively) were monitored from C0 (beginning of the surgery) to C40 (40 min after the beginning of the surgery) in both groups. Significant differences were found in SAP, DAP and MAP from C10 to C35, with lower values in Group 2 (Figure 10, Figure 11 and Figure 12).

### 3.5. Pain Scale

Buffaloes of both groups were monitored at 30, 60 and 90 min and then at 6-, 12- and 24-h intervals after surgery. No significant difference in the median BOTUCATU score was found between the groups (Table 6).

## 4. Discussion

### 4.1. Aims

The aim of this study was to define an appropriate anaesthetic protocol that can be used to perform standard surgical procedures on buffalo calves. Because the anatomical and physiological characteristics of cows are similar to those of buffaloes, buffaloes are often considered and managed as cattle, but the impact of interspecies differences on the pharmacokinetics and pharmacodynamics of drugs should be considered [10]. We investigated the effect of two different intramuscular doses of xylazine for premedication, followed by ketamine induction, in water buffalo calves undergoing isoflurane maintenance anaesthesia for routine castration surgery. In our experimental study, both protocols provided consistent sedation and were found to be safe in buffaloes. Faster and more satisfactory sedation was recorded when xylazine was administered at 0.15 mg/kg bw I.M. compared to the lower dose.

A combination of physical restraint, sedation and local or regional anaesthesia is mostly used for basic surgical procedures in large ruminants [24]. In more complex surgeries, general anaesthesia is preferred because it maximises effectiveness and safety; nevertheless, general anaesthesia can lead to complications, such as regurgitation, tympanism and aspiration pneumonia [18,25,26,27]. Moreover, general anaesthesia can suppress autonomic reflex activities and consequently induce dose-dependent cardiovascular and respiratory depression [28]. To reduce the risk of these complications, inhaled anaesthesia is recommended, as it allows quicker changes in anaesthetic depth and lesser risk of overdosing compared to injectable anaesthesia [24]. In this study, we selected isoflurane, as it offers faster recovery from anaesthesia and less cardiovascular, respiratory and haemodynamic depression than halothane in water buffaloes [24]. However, isoflurane, like other inhaled agents, has little or no analgesic effect, and thus, the incorporation of intravenous drugs into the anaesthetic protocol is mandatory to help decrease the concentration of isoflurane required for the maintenance of anaesthesia [28]. The use of sedatives prior to the induction of anaesthesia not only improves the quality of anaesthesia but also decreases the adverse effects of each individual drug used [29]. Alpha_2_-adrenoceptor agonist agents have sedative, tranquilising, analgesic and muscle relaxant properties [12,13]. Detomidine at 40 µg/kg intramuscularly provides satisfactory sedation with marked ataxia and sternal recumbency in buffalo calves but induces marked bradycardia for the first hour after injection [30]. Medetomidine (10 µg/kg intravenously) and romifidine (50 µg/kg intravenously) have been proven to cause moderate sedation and a mild degree of muscle relaxation and analgesia in buffalo calves and are considered safe as preanesthetic agents [13]. However, the use of neither drug is allowed in ruminants [11]. We decided to use xylazine rather than medetomidine, detomidine or romifidine, as its use is allowed in ruminants. We selected different doses of xylazine based on an earlier study by Alshara and colleagues [16]. The results of their study suggest that xylazine, when administered intramuscularly, causes sedation and moderate analgesia and, therefore, can be used as a pre-anaesthetic drug in buffaloes [16].

### 4.2. Interpretation of Clinical Observations

*Sedation* and *ataxia* were evaluated based on the modified scheme from Valverde and colleagues [17]. In Group 2 at T2, buffalos started to show a moderate response to external stimuli and higher levels of sedation, with a loss of response to stimuli occurring at T15. Conversely, in Group 1, buffaloes reached the same level of sedation between T10 and T15. Both groups reached a stable peak before T40.

Although no significant difference was observed between the median sedation values of the two groups from T0 to T40, we can assume that in the initial stages, faster effective sedation can be obtained with a higher dose of xylazine. This theory is also supported by the results of the ataxia scores. In Group 2, indeed, statistically higher ataxia scores were found at T2 and T5. Moreover, a significant peak, indicating that most buffalo calves had already adopted the sternal positions, was seen in Group 2 at T10, while in Group I, this peak was reached at T20–T25. Additionally, clinical observation of the degree of sedation evaluated based on the modified scheme from Singh and colleagues suggested that good sedation was provided in both groups, with faster and more pronounced results in Group 2 [18]. As shown in Table 5 and Table 6, the median values increased in both groups as a function of time.

The *depth of anaesthesia* score was significantly higher in Group 2 at T0, T5 and T10. After that time, the depth of anaesthesia was similar in both groups until T40. A good sedation score was reached in Group 1 only after T15, while in Group 2, a good sedation score was reached at T5, showing more effective and faster sedation in Group 2. Regarding *responses to acoustic and tactile stimuli,* an excellent and progressive decrease in the perception of stimuli was observed in both groups. Statistically higher values were reached in Group 2 at T5 and T10, suggesting a faster and more pronounced effect of the protocol used in Group 2 in the initial stages of anaesthesia. A significant difference was also found in the *muscle relaxation* score between groups from T2 to T10, with Group 2 presenting higher values. The higher dose of xylazine used in Group 2 thus guaranteed a quicker and more stable myorelaxant effect, especially in the early stages of anaesthesia.

These results are also supported by the faster and easier jugular vein catheterization and intubation in Group 2. Calves of both groups were evaluated ultrasonographically in three portions of the neck to choose the best place to insert the venous catheter and allow easy catheterisation of the vein. However, as shown in Table 2, the mean catheterization time in Group 2 was significantly shorter, and a significantly lower number of attempts was necessary to perform the catheterisation. Moreover, the catheterisation of the jugular vein was performed in all the buffalos of Group 2, whereas in Group 1, catheterisation failed in four buffalos, indicating a more effective sedation in Group 2 that allowed easier catheterisation. Additionally, the mean intubation time was significantly lower in Group 2, indicating a fast and adequate level of sedation compared to Group 1. If there is not good sedation, indeed, muscle relaxation is not sufficient, leading to difficult and longer intubation.

The effectiveness of the anaesthetic protocol used in Group 2 is also supported by the fact that buffalo calves in Group 2 were significantly smaller than those in Group 1. Animals with a lower weight are indeed more difficult to intubate due to the difficulty of correctly visualising the larynx. However, buffaloes in Group 2, despite their smaller size, were intubated faster than those in Group 1.

The number of top-ups was also recorded. Although we expected to observe significant differences between the groups, both groups required the same number of xylazine top-ups; although a greater use of ketamine top-ups was necessary in Group 1, this difference was not statistically significant. The duration of anaesthesia and the duration of the surgical procedure were also similar between groups. Group 2 had a slightly shorter duration, but this difference was not statistically significant, indicating that the higher dose of xylazine used had less influence in the maintenance phase than in the early stages of sedation.

The time to extubation and sternal recumbency, the number of attempts and the time to the standing position were evaluated in both groups. In accordance with previous literature, recovery times with xylazine administered intramuscularly are prolonged [18]. The buffalo calves in Group 2 experienced a longer recovery than those in Group 1, but these differences were not statistically significant. When considering the final recovery score, both groups exhibited excellent recovery. No significant difference in the final recovery score between groups was observed, although this score was higher in Group 2.

### 4.3. Physiological and Hemodynamic Considerations

No intraoperative complications were recorded in Group 1. In Group 2, one buffalo presented reflux after general anaesthesia was started. To reduce the risks associated with potential anaesthetic complications, the buffaloes were fasted for 12 h, and water was withheld for 8 h prior to anaesthesia. Fasting represents a means of preventing the onset of tympanism and regurgitation and decreasing the mass of the rumen content [16]. Even with these precautions, 3/10 buffaloes in both groups showed mild regurgitation, and 3/10 buffaloes in Group 2 showed mild tympanism. Furthermore, a buffalo from Group 2 died at 24 h postoperatively because of tympanism, probably due to inadequate fasting prior surgery.

Xylazine is widely used in cattle, but in buffaloes, high doses are associated with increased salivation [15]. This effect on salivary glands has been attributed both to increased secretion through alpha-1-adrenergic receptors and to a decreased swallowing reflex [18]. In our study, sialorrhea was observed in both groups but to a greater extent in Group 2, especially in the early stages of anaesthesia (T5, T10 and T15). Therefore, the use of a higher dose of xylazine increased saliva production, enhancing the risk of aspiration pneumonia. Endotracheal intubation with an adequate ET tube properly cuffed is thus mandatory to provide a safe airway and prevent the aspiration of salivary contents.

Xylazine in buffaloes also causes hypotension and bradycardia [15,31]. Bradycardia is caused by the effects of the drug on the central and autonomic nervous systems and the compensatory response to cardiovascular depression [18]. The inhibition of sympathetic tone indeed leads to reduced blood pressure and cardiac output, and the initial vasoconstriction activates the parasympathetic response, resulting in bradycardia [18]. Hypotension is attributed to bradycardia and vasodilation by the stimulation of central alpha-2 adrenoceptors and enhanced parasympathetic activity [18]. In our study, both groups showed a similar decrease in HR. Although the xylazine dose was higher in Group 2, there was no significant difference between the two groups, except for HR from T30 to T40. Hypotension was observed in both groups in our study. Significant differences were found in SAP, DAP and MAP from C10 to C35, with lower values in Group 2. The higher dose of xylazine used in Group 2 thus resulted in a more pronounced hypotension, especially in the central stages of the surgical procedure. A decrease in RR was also recorded in both groups during sedation. Respiratory depression associated with xylazine is caused by CNS depression produced by alpha-2 adrenoceptor stimulation [18]. Although the xylazine dose was higher in Group 2, no significant difference in RR was found between groups. Blood gas analysis was not available for all patients, and unfortunately, it was not possible to have all the available samples retrieved at the same time after the induction of anaesthesia, so data were not analysed. The lack of blood gas analysis data can be considered a limitation of the study.

### 4.4. Interpretation of Pain Scale Results

An NSAID was not administered before sedation to limit any influence of the response to xylazine administration on either group. However, all calves received flunixin meglumine just before the beginning of surgery. During surgery, an intrafunicular block was performed to reduce intra- and postoperative pain. Vasodilation associated with lidocaine increases the risk of haemorrhage, but no significant blood loss was found in castrated buffalo calves in our study; however, painful attitudes after surgery were seen. We monitored the development of pain and attitudes in our buffalo calves according to the UNESP-Botucatu unidimensional composite pain scale for assessing postoperative pain in cattle [23]. Patients were monitored at 30, 60 and 90 min and then at 6-, 12- and 24-h intervals. During the first hour after castration, some buffalo calves showed typical attitudes of pain. In Group 1, a median score of 1 was never reached or exceeded, while in Group 2, a median score of 2 was never reached or exceeded. Therefore, for most buffalo calves, there was an excellent recovery after surgery in both groups, with no statistically significant difference reported between the groups.

In our experimental study, the number of calves was adequate to perform a good statistical analysis. However, furthers analysis of blood gas data are required to adequately evaluate cardiocirculatory and respiratory functions in our experimental protocol, despite not suitable under field conditions.

## 5. Conclusions

Based on the results obtained, we can conclude that both protocols are appropriate for buffalo castration, but the higher dose protocol is recommended for buffalo orchiectomy. The higher dose has more satisfactory effects in the early preoperative stages, showing a good loss of reflexes and muscle relaxation. This fast and excellent sedative effect led to easier jugular vein catheterisation in Group 2, also reducing the time needed for intubation. Nevertheless, calves in Group 2 showed a more pronounced hypotension and a higher risk of tympanism compared to calves in Group 1. An adequate fasting period to reduce the risk of tympanism and regurgitation is recommended.

## Figures and Tables

**Table 1:** Parameters used for clinical monitoring of anaesthesia in our study (modified from Valverde A et al., 1989 and from Singh GD et al., 2013) [17,18].

Parameters	Score	Interpretation
Ataxia	0	Absent (no ataxia)
	1	Mild (knuckling of the fetlocks)
	2	Moderate (crossing of the hindlimbs)
	3	Strong (attempting or assuming recumbency)
Sedation	0	No sedation (alert, eyes open)
	1	Mild (drooping of eyelids)
	2	Moderate (lowering the head, minimum to moderate reaction to noise nociception and handling)
	3	Deep (no reaction to noise nociception and handling)
Depth of anaesthesia	0	No sedation (alert, eyes open)
	1	Mild (drooping of eyelids, mild sensory and motor deficit)
	2	Moderate (drooping of eyelids, moderate sensory and motor deficit)
	3	Strong (drooping of eyelids, severe sensory and motor deficit)
Muscle relaxation	1	Absent (tightly closed jaws and stiff limbs, no flaccidity of the abdomen)
	2	Mild (moderate resistance to the opening of the jaws and bending of the limbs, no flaccidity of the abdomen)
	3	Moderate (mild resistance to the opening of the jaws and bending of the limbs, no flaccidity of the abdomen)
	4	Excellent (no resistance to the opening of the jaws, bending of the limbs and flaccid abdomen)
Response to acoustic stimulus	1	Strong reaction
	2	Weak response
	3	Occasional response
	4	No response
Response to tactile stimulus	1	No analgesia (strong reaction)
	2	Mild analgesia (weak response)
	3	Moderate analgesia (occasional response)
	4	Excellent analgesia (no response)
Eyelid reflex	0	- Completely abolished reflexes
	1	+ Mildly abolished reflexes
	2	++ Moderately abolished reflexes
	3	+++ Slightly intact reflexes
	4	++++ Intact reflexes
Salivation	0	- Absent
	1	+ Mild
	2	++ Moderate
	3	+++ Extensive
	4	++++ Profuse
Regurgitation/tympanism/	No	Absent
reflux	Yes	Present

**Table 2:** Age, body weight, catheterisation time, catheterisation attempts, intubation time, ET number and number of top-ups in Groups 1 and 2. Values are expressed as the mean ± SD (SD: standard deviation; ET: endotracheal tube). Statistically different values are highlighted in yellow.

	Group 1	Group 2
Age (months)	3.15 ± 1.31	2.3 ± 0.25
Body weight (kg)	176.50 ± 43.64	102.20 ± 20.31
Catheterisation time (minutes)	13.00 ± 11.17	5.01 ± 4.50
Catheterisation attempts (number)	3.14 ± 1.17	1.5 ± 1.26
Intubation (minutes)	7.40 ± 11.36	2.50 ± 2.01
#ET tube	16.20 ± 2.39	12.80 ± 1.03
Top-ups of xylazine	0.80 ± 0.63	0.90 ± 0.99
Top-ups of ketamine	1.20 ± 2.57	0.30 ± 0.48

**Table 3:** Mean recovery values in Groups 1 and 2. Values are expressed as the mean ± SD (SD: standard deviation).

Group	Anaesthesia Time (min)	Surgical Time (min)	Time to Extubation (min)	Time to Sternal Recumbency (min)	Time to Quadrupedal Station (min)	Recovery Score
Group 1	39.2 ± 13.56	23.60 ± 11.68	7.20 ± 5.95	8.40 ± 6.07	18.70 ± 20.87	1.7 ± 1.05
Group 2	30.9 ± 5.52	15.50 ± 3.77	10.80 ± 4.31	13.00 ± 4.64	20.90 ± 15.63	2.30 ± 1.25

**Table 4:** Mean HR and RR in Groups 1 and 2. Values are expressed as the mean ± SD (SD: standard deviation). Different letters in the same column indicate significantly different results (a, ab: *p* < 0.05).

	Group	T0	T2	T5	T10	T15	T20	T25	T30	T35	T40
HR	1	68.40 ± 19.17	47.60 ± 5.39	42.80 ± 5.67	40.00 ± 5.65	36.60 ± 6.39	36.00 ± 5.65	36.20 ± 6.89	34.00 ± 4.32 ^a^	33.2 ± 3.42 ^a^	38.5 ± 7.09 ^ab^
	2	57.40 ± 7.77	44.80 ± 5.20	42.80 ± 4.23	40.00 ± 4.71	36.00 ± 9.61	38.40 ± 8.26	42.20 ± 8.56	41.70 ± 5.88 ^a^	41.00 ± 5.45 ^a^	48.00 ± 15.52 ^ab^
RR	1	22.2 ± 6.49	23 ± 8.80	19.7 ± 5.61	17.1 ± 3.95	14.3 ± 6.21	16.6 ± 9.66	14.5 ± 12.60	13.1 ± 5.80	15.3 ± 11.05	9.00 ± 4.29
	2	19.80 ± 4.26	19.40 ± 5.66	19.60 ± 5.56	17.60 ± 6.58	15.80 ± 5.99	16.60 ± 4.00	14.70 ± 5.85	16.00 ± 12.50	18.30 ± 15.76	15.00 ± 10.98

**Table 5:** Evaluation of sedation from T0 to T35 compared in Group 1 and Group2. Values are expressed as the median ± range. Different letters in the same column indicate significantly different results (a, ab, b, bc, c: *p* < 0.05).

	Group	T0	T2	T5	T10	T15	T20	T25	T30	T35
Depth of anaesthesia	1	0 ^c^	0.40 ± 0.51	0.90 ± 0.56 ^ab^	1.60 ± 0.69 ^a^	2.50 ± 0.52	2.60 ± 0.51	2.70 ± 0.48	2.90 ± 0.31	2.80 ± 0.42
	2	0.40 ± 0.51 ^c^	0.70 ± 0.67	1.50 ± 0.70 ^ab^	2.00 ± 0.94 ^a^	2.30 ± 0.94	2.50 ± 0.97	2.50 ± 0.97	2.60 ± 0.96	2.60 ± 0.96
Salivation	1	0.30 ± 0.48	0.30 ± 0.48	0.60 ± 0.5 ^bc^	0.80 ± 0.63 ^ab^	0.90 ± 0.56 ^b^	0.80 ± 0.42	0.70 ± 0.48	0.70 ± 0.48	0.70 ± 0.48
	2	0.3 ± 0.48	0.70 ± 0.48	1.1 ± 0.73 ^bc^	1.50 ± 0.70 ^ab^	1.50 ± 0.70 ^b^	1.20 ± 0.78	1.10 ± 0.87	1.00 ± 0.81	1.00 ± 0.81
Sedation acoustic stimulus	1	1	1.10 ± 0.31	1.40 ± 0.69 ^ab^	2.80 ± 1.13 ^a^	3.60 ± 0.84	3.30 ± 0.94	3.50 ± 1.08	3.50 ± 0.97	3.70 ± 0.67
	2	1	1.60 ± 0.69	2.30 ± 0.82 ^ab^	3.50 ± 0.70 ^a^	3.70 ± 0.67	3.60 ± 0.69	3.70 ± 0.67	3.70 ± 0.67	3.70 ± 0.67
Sedation tactile stimulus	1	1	1.20 ± 0.42	1.60 ± 0.84 ^bc^	2.90 ± 0.99	3.50 ± 0.84	3.30 ± 0.94	3.60 ± 0.84	3.80 ± 0.63	3.80 ± 0.42
	2	1	1.50 ± 0.52	2.20 ± 0.78 ^bc^	3.20 ± 0.91	3.60 ± 0.69	3.60 ± 0.69	3.60 ± 0.69	3.60 ± 0.69	3.60 ± 0.69
Muscle relaxation	1	1	1 ^ab^	1.50 ± 0.52 ^bc^	2.10 ± 0.87 ^a^	2.60 ± 0.96	2.80 ± 1.03	3.40 ± 0.69	3.50 ± 0.52	3.40 ± 0.69
	2	1	1.50 ± 0.52 ^ab^	2.00 ± 0.81 ^bc^	2.90 ± 0.99 ^a^	3.20 ± 0.91	3.20 ± 0.91	3.30 ± 0.94	3.50 ± 0.97	3.50 ± 0.97
Eyelid reflex	1	4	4	3.50 ± 0.52	1.70 ± 1.15	1.60 ± 1.17	1.40 ± 0.84	1.30 ± 0.82	1.40 ± 0.84	1.50 ± 0.97
	2	4	3.7 ± 0.48	3.30 ± 0.82	2.00 ± 1.33	1.70 ± 1.56	1.90 ± 1.44	1.80 ± 1.54	1.40 ± 1.42	1.50 ± 1.43
Reflux/regurgitation/ tympanism	1	NO	NO	NO	NO	NO	NO	NO	NO	NO
	2	NO	NO	NO	NO	NO	NO	NO	1 buffalo	NO

**Table 6:** Median BOTUCATU score compared between Group 1 and Group 2. “Score” values are expressed as the median ± range. “Attitude” values are expressed as a letter, corresponding to a particular behaviour, and a number, corresponding to the number of buffaloes presenting that behaviour (A: wagging the tail abruptly and repeatedly; E: hindlimbs extended caudally when in the standing posture; F: head below the spinal column; G: lying down in ventral recumbency with full or partial extension of one or both hindlimbs; H: lying down with the head on/close to the ground).

Groups		30”	60”	90”	P6H	12H	24H
Group 1	Score	0.90 ± 1.59	0.90 ± 1.66	0.40 ± 0.69	0.40 ± 1.26	0	0
	Attitude	A (1/10) G (1/10)	F (2/10) G (1/10)	F (2/10)	H (1/10)	-	-
Group 2	Score	1.4 ± 2.06	1.5 ± 2.27	0.6 ± 1.07	0.1 ± 0.31	0.6 ± 1.89	0.2 ± 0.63
	Attitude	F (2/10) H (1/10)	F (2/10) H (1/10)	F (1/10)	-	E (1/10)	-

## Data Availability

Data are available on request due to privacy restriction. The data presented in this study are available on request from the corresponding author.
